# Ubiquitin-specific peptidase 14 maintains estrogen receptor α stability *via* its deubiquitination activity in endometrial cancer

**DOI:** 10.1016/j.jbc.2022.102734

**Published:** 2022-11-22

**Authors:** Yingjie Su, Kai Zeng, Shuchang Liu, Yi Wu, Chunyu Wang, Shengli Wang, Lin Lin, Renlong Zou, Ge Sun, Ruina Luan, Baosheng Zhou, Yu Bai, Jumin Niu, Yi Zhang, Yue Zhao

**Affiliations:** 1 Department of Cell Biology, Key laboratory of Cell Biology, Ministry of Public Health, and Key Laboratory of Medical Cell Biology, Ministry of Education, School of Life Sciences, China Medical University, Shenyang City, Liaoning Province, China; 2 Department of Gynecology, The First Hospital of China Medical University, Shenyang City, Liaoning Province, China; 3 Department of Gynecology, The Fourth Affiliated Hospital of China Medical University, Shenyang City, Liaoning Province, China; 4 Department of Pathogenic Biology, Shenyang Medical College, Shenyang, Liaoning, China; 5 Department of Obstetrics and Gynecology, Shenyang Women's and Children's Hospital, Shenyang, Liaoning, China

**Keywords:** USP14, ERα, deubiquitination activity, USP14 inhibitor, endometrial cancer, AR, androgen receptor, BCa, breast cancer, ChIP, chromatin immunoprecipitation, co-IP, coimmunoprecipitation, E2, unopposed estrogen, EC, endometrial cancer, ERα, estrogen receptor α, ERE, estrogen-response element, HA, hemagglutinin, HEC-1A, human endometrial adenocarcinoma 1A cell, IHC, immunohistochemical, K48, lysine 48, K63, lysine 63, qPCR, quantitative PCR, shUSP14, shRNA against USP14, USP14, ubiquitin-specific peptidase 14

## Abstract

USP14 deubiquitinates ERα to maintain its stability in ECEndometrial cancer (EC) is one of the common gynecological malignancies of which the incidence has been rising for decades. It is considered that continuously unopposed estrogen exposure is the main risk factor for EC initiation. Thus, exploring the modulation of estrogen/estrogen receptor α (ERα) signaling pathway in EC would be helpful to well understand the mechanism of EC development and find the potential target for EC therapy. Ubiquitin-specific peptidase 14 (USP14), a member of the proteasome-associated deubiquitinating enzyme family, plays a crucial role in a series of tumors. However, the function of USP14 in EC is still elusive. Here, our results have demonstrated that USP14 is highly expressed in EC tissues compared with that in normal endometrial tissues, and higher expression of USP14 is positively correlated with poor prognosis. Moreover, USP14 maintains ERα stability through its deubiquitination activity. Our results further demonstrate that USP14 depletion decreases the expression of ERα-regulated genes in EC-derived cell lines. Moreover, knockdown of USP14 or USP14-specific inhibitor treatment significantly suppresses cell growth and migration in EC cell lines or in mice. We further provide the evidence to show that the effect of USP14 on EC cell growth, if not all, at least is partially related to ERα pathway. Our study provides new sights for USP14 to be a potential therapeutic target for the treatment of EC, especially for EC patients with fertility preservation needs.

Endometrial cancer (EC) is one of the gynecological malignancies, with an increasing incidence in the world (1, 2). It has been reported that EC patients who are at advanced stage or suffer from recurrence have poor prognosis (3). According to the updated statistics, there are about 66,570 new cases and 12,940 deaths in the United States in 2021 (4), indicating an increase in the morbidity and mortality of EC. Importantly, because of the younger age of EC onset, the treatment strategies with fertility preservation are critical and challenging. Therefore, it is necessary to understand the molecular mechanism of EC development and explore more effective and therapeutic target for EC treatment.

In consideration of hormonal dependence in EC, it is divided into two types, including type I hormone dependent and type II hormone independent (5). Type I accounts for 80% of the EC cases, with the feature of positive estrogen receptor α (ERα) expression (6). It is generally known that long-term unopposed estrogen (E2) exposure is the main risk factor for type I EC, indicating that E2–ERα signaling pathway plays a significant role in EC development and progression. Previous studies have shown that ERα, as a transcription factor, is involved in the pathogenic process in breast cancer (BCa) (7). A number of coregulators involved in the modulation of ERα activity promote tumorigenesis and contribute to endocrine therapy resistance in BCa (8, 9, 10). However, the regulation of ERα signaling pathway and its underlying biological function in EC progression are still largely unknown.

Ubiquitin-specific protease 14 (USP14), as one of the proteasome-associated deubiquitinase, exerts important roles in several carcinomas (11, 12). It has been reported that USP14 appears to have quite different functions in modulating intracellular proteolytic degradation involving in both maintenance of protein stability and protein degradation (13, 14). Ubp6, a yeast homolog of USP14, participates in delaying protein degradation to lead to protein accumulation (15). Studies have been shown that USP14 stabilizes androgen receptor (AR) protein through its deubiquitination activity. USP14 can also promote cell growth and inhibit cell apoptosis in AR-positive ERα-negative BCa (16). USP14 interacts with murine double minute 2 and stabilizes murine double minute 2, leading to tumor progression in cervical cancer (17). USP14 regulates cisplatin resistance in ovarian cancer through stabilizing BCL6 protein (18). However, the functional analysis of USP14 in EC is still largely elusive.

In this study, we have demonstrated that USP14 is highly expressed in EC clinical samples compared with that in noncarcinoma endometrial tissues. The higher expression level of USP14 is positively correlated with the poor prognosis of EC. Our results also provide the evidence that USP14 is involved in the maintenance of ERα stability through its deubiquitinating activity. USP14 deubiquitinates ERα at lysine 48 (K48)-linked ubiquitination. In addition, depletion of USP14 downregulates ERα-induced gene transcription. Knockdown of USP14 decreases the recruitment of ERα to estrogen-response element (ERE) of *c-Myc*, which is one of the putative ERα target genes. Furthermore, USP14 depletion or USP14-specific inhibitor attenuates cell growth and migration in EC-derived cell lines or in mice. Taken together, our study may provide a potential therapeutic target for the treatment of EC, especially for EC patients with fertility preservation needs.

## Results

### USP14 is highly expressed in EC tissues, and the higher expression of USP14 is positively correlated with the poor prognosis of EC patients

**Figure 1:**
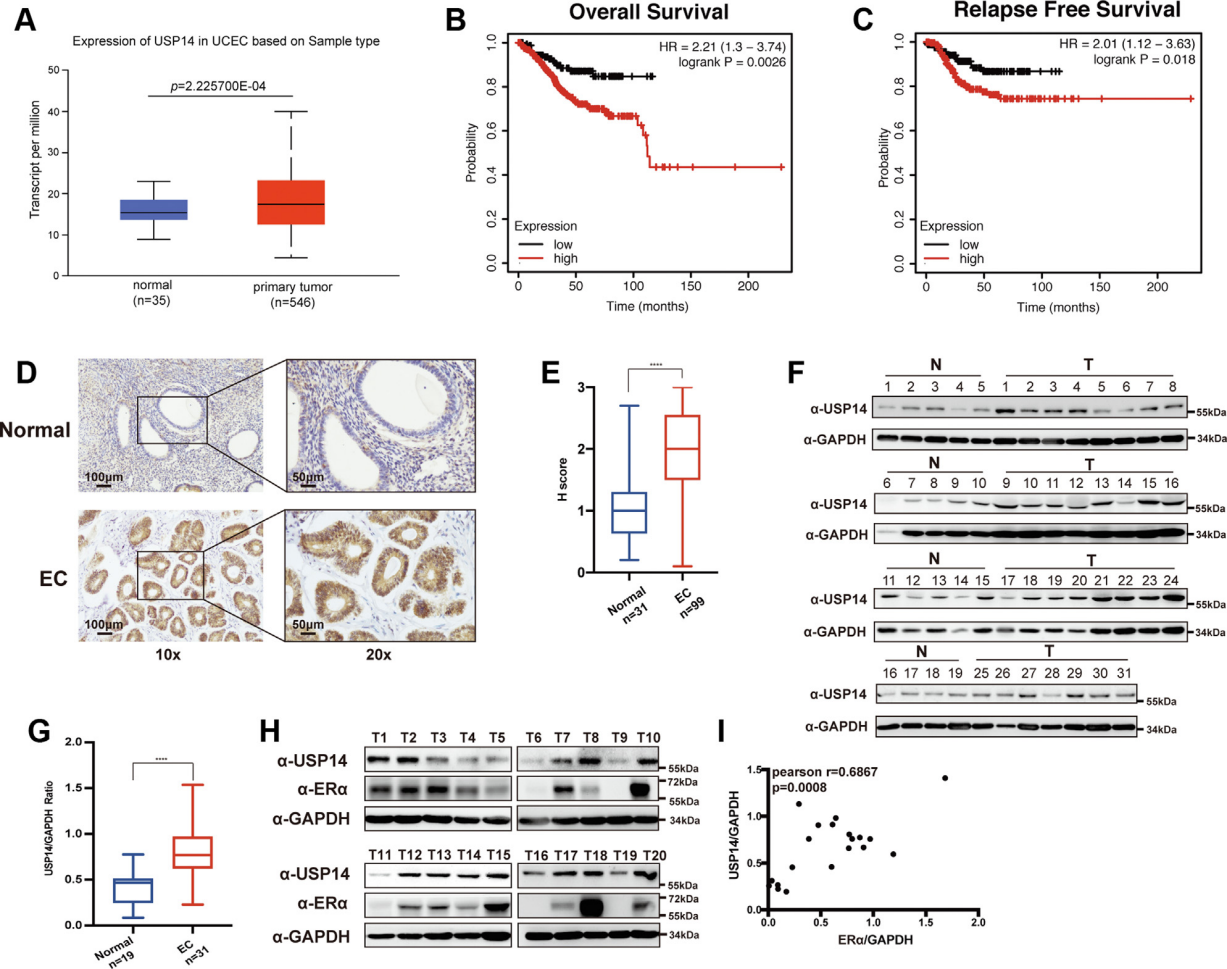
**USP14 is overexpressed in EC and negatively associated with the prognosis.***A*, USP14 was overexpressed in EC patients according to the UALCAN database. *B* and *C*, Kaplan–Meier analysis was used to detect the overall survival and relapse-free survival for the EC patients based on the Cancer Genome Atlas. *D* and *E*, immunohistochemical assay was used to detect the USP14 expression in normal tissues and cancerous tissues. Magnification: 10×/20×: the scale bars represent 100 μm/50 μm. *F* and *G*, expression level of USP14 was detected by Western blot and quantitated with ImageJ in the normal tissues (n = 19) and cancerous tissues (n = 31). *H* and *I*, Western blot was used to determine the expression of USP14 and ERα in 20 EC tissues. Pearson correlation test showed the statistical significance between USP14 and ERα. EC, endometrial cancer; ERα, estrogen receptor α; USP14, ubiquitin-specific peptidase 14.

Previous studies have shown that USP14 is highly expressed in a variety of malignancies and plays a critical role in tumor progression (19). However, the expression level and functions of USP14 in EC remain poorly defined. To this end, we then analyzed the expression of USP14 in EC tissues and normal endometrial tissues using UALCAN (http://ualcan.path.uab.edu/index.html). The results demonstrated that USP14 has a higher expression in EC tissues (Fig. 1*A*) (20). We then analyzed the expression level of USP14 at different clinical stages and histological subtypes. The results indicated that there was no obvious difference among the stages and various histological subtypes (Fig. S1, *A* and *B*). To further investigate whether there was a correlation between USP14 and the clinical outcomes of the patients, we performed an analysis through Kaplan–Meier plotter based on the Cancer Genome Atlas (21); the results showed that the aberrant expression of USP14 was negatively associated with the overall survival and relapse-free survival (Fig. 1, *B* and *C*).

We then detected the expression of USP14 in the collected 31 normal tissues and 99 EC tissues from the First Hospital of China Medical University. Hereafter, Western blot was conducted to further determine the expression of USP14 in 19 noncarcinoma tissues and 31 EC tissues. The results from immunohistochemistry assay or Western blotting showed that the expression level of USP14 was significantly higher in the EC tissues compared with that in the noncarcinomous tissues (Fig. 1, *D–G*). Having established that ERα plays a significant role in EC progression, we then examined the corresponding expression of USP14 and ERα in 20 EC samples. Our results showed that the expression of USP14 was positively correlated with that of ERα in EC samples (Fig. 1, *H* and *I*).

### USP14 participates in the maintenance of ERα stability

**Figure 2:**
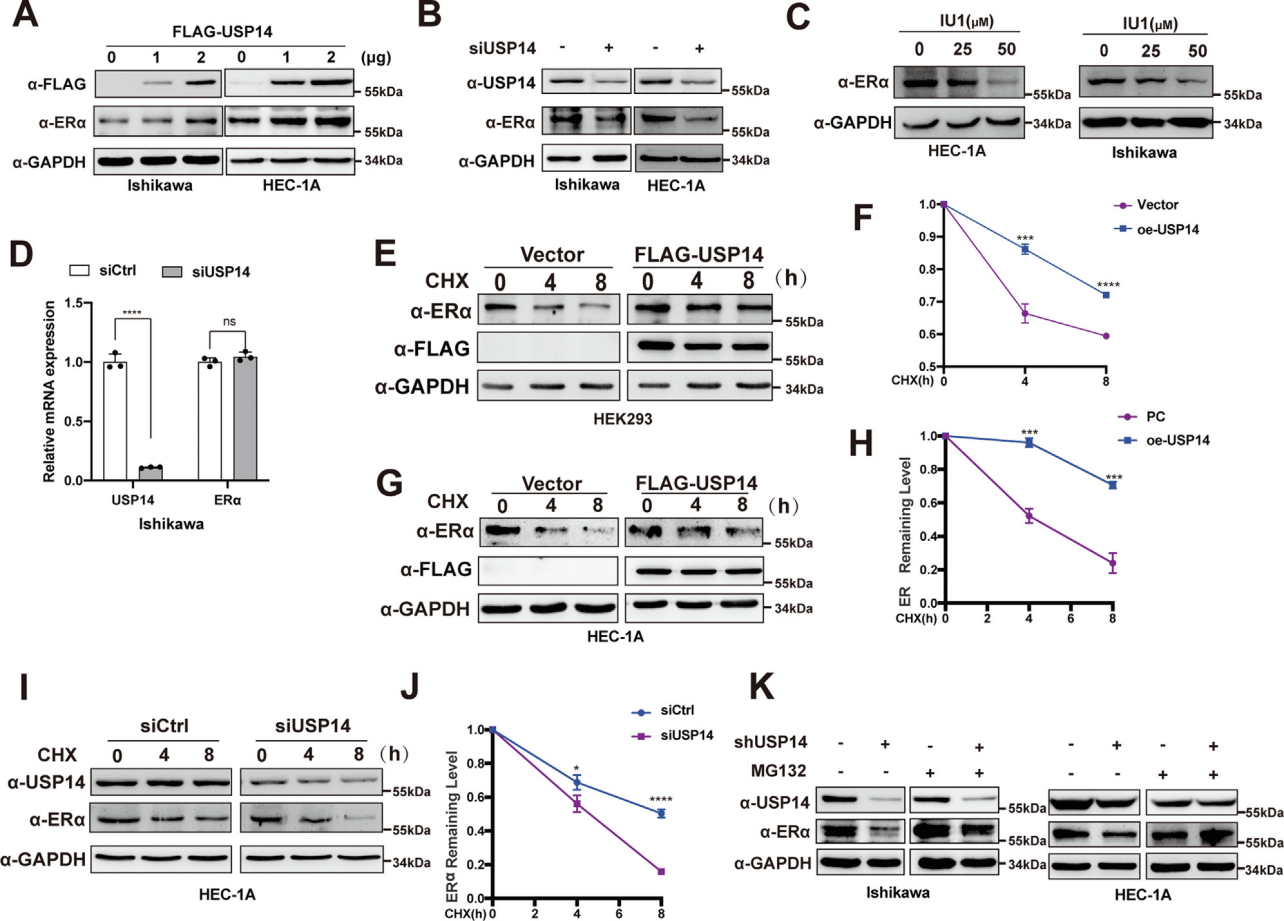
**USP14 participates in the maintenance of ERα stability.***A*, overexpression of USP14 increases the ERα protein level in a dose-dependent manner in both Ishikawa and HEC-1A cell lines. *B*, depletion of USP14 in EC cell lines can decrease the ERα protein level. *C*, protein lysate was collected with indicated concentration of USP14-specific inhibitor (IU1) for 48 h in both EC cell lines. *D*, total RNA of Ishikawa cells transfected with siCtrl or siUSP14 was used to analyze the *ESR1* mRNA level. *E* and *F*, HEK293 was transfected with ERα and USP14. The cell was treated with 50 mg/ml cycloheximide (CHX) for 0, 4, and 8 h, and then the ERα protein level was detected with Western blot. *G* and *H*, HEC-1A cells were transfected with USP14 and treated with 50 mg/ml cycloheximide (CHX) for indicated time points. The remaining ERα protein level was quantitated by ImageJ. *I* and *J*, HEC-1A cells were transfected with siCtrl or siUSP14 and treated with CHX as HEK293. The ERα protein level was calculated as described above. *K*, depletion of USP14 decreased ERα protein level in both Ishikawa cells and HEC-1A cells. The cell lysate was treated with MG132 (5 μM) for 6 h before collected. ∗*p* < 0.05, ∗∗*p* < 0.01, ∗∗∗*p* < 0.001, and ∗∗∗∗*p* < 0.0001. EC, endometrial cancer; HEK293, human embryonic kidney 293 cell line; ERα, estrogen receptor α; USP14, ubiquitin-specific peptidase 14.

Having known the correlation between the expression of USP14 and ERα, we then turned to investigate the underlying mechanism between them. We measured the expression of ERα following ectopic expression of USP14, which indicated USP14 could affect ERα at protein level in a dose-dependent manner (Figs. 2*A* and S2*A*). Then, in the EC cell lines with USP14 depletion or treated with indicated IU1, a specific inhibitor of USP14 (22), a significant decrease in ERα expression was noted at the protein level but not at the mRNA level (Figs. 2, *B–D* and S2, *B* and *C*). These results suggested that USP14 affected ERα by inhibiting its degradation but not regulating its transcription. To further verify this opinion, we treated the cells with protein synthesis inhibitor, cycloheximide to determine the influence of USP14 on ERα in human embryonic kidney 293 and human endometrial adenocarcinoma 1A (HEC-1A) cells. The results demonstrated that USP14 overexpression retarded ERα degradation, whereas inhibition of USP14 accelerated ERα degradation (Fig. 2, *E–J*). In addition, in the presence of the proteasome inhibitor MG132, the increase of ERα by USP14 overexpression was reversed in both Ishikawa and HEC-1A cell lines (Figs. 2, *K* and S2*D*). Taken together, these data suggested that USP14 might be involved in the maintenance of ERα stability *via* inhibiting the proteasome pathway in EC cell lines.

### USP14 interacts with ERα and deubiquitinates ERα with K48-linkage

**Figure 3:**
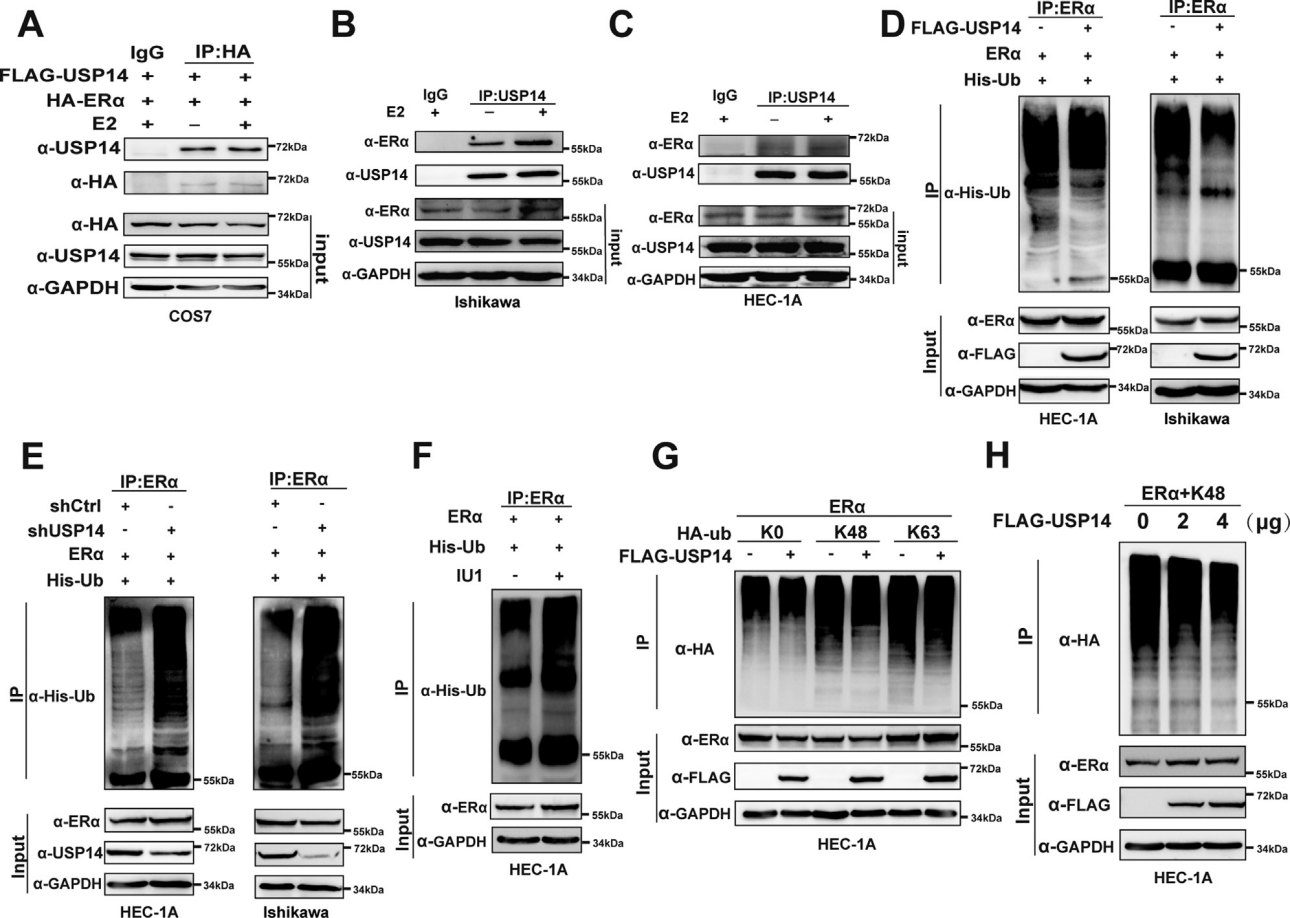
**USP14 interacts with ERα and deubiquitinates ERα *via* K48-linkage.***A*–*C*, USP14 interacted with ERα. Coimmunoprecipitation (Co-IP) assay was performed in COS7 cells and EC cell lines with indicated antibodies. *D* and *E*, IP-based ubiquitination assays were conducted in both EC cell lines. Overexpression or depletion of USP14 could decrease or increase ERα ubiquitination level in EC cell lines. *F*, HEC-1A cells were treated with indicated IU1 for 48 h. IP-based ubiquitination assays were performed with ERα antibodies. *G* and *H*, HEC-1A cells were transfected with expression plasmid encoding FLAG-USP14 and ERα together with HA-tagged ubiquitin mutants, including K0 (lysineless), K48 (only K48-linked Ub), and K63 (only K63-linked Ub) as indicated. The cells were treated with MG132 (5 μM) before collected. The cell lysate was immunoprecipitated with anti-ERα and immunoblotted with anti-HA. EC, endometrial cancer; ERα, estrogen receptor α; HA, hemagglutinin; USP14, ubiquitin-specific peptidase 14.

We further turn to determine whether USP14 interacts with ERα. The expression plasmids of FLAG-tagged-USP14 and hemagglutinin (HA)-tagged-ERα were then transfected to perform coimmunoprecipitation (co-IP) experiments in COS7 cell lines. The results demonstrated that USP14 interacted with ERα with or without E2 treatment (Fig. 3*A*). To further confirm these results, we performed co-IP in EC cell lines (Ishikawa and HEC-1A cell lines); the results suggested an endogenous interaction between USP14 and ERα in EC-derived cell lines (Fig. 3, *B* and *C*). Because of the deubiquitination activity of USP14, we wonder whether USP14 can participate in influence of ERα ubiquitination. Immunoprecipitation-based ubiquitination assays were then performed as indicated. The results demonstrated that the ectopic expression of USP14 apparently reduced the level of ERα ubiquitination in EC cell lines (Fig. 3*D*). Moreover, USP14 depletion or USP14 inhibitor treatment significantly increased ERα ubiquitination (Fig. 3, *E* and *F*). Thus, these results indicated that USP14 participated in deubiquitination of ERα to maintain stabilization of ERα protein.

It has been well established that different polyubiquitination types of protein exert various functions, of which K48- and lysine 63 (K63)-linked polyubiquitin chain represents a signal for proteasomal degradation (23, 24). We turned to determine which type of polyubiquitin chain on ERα was affected by USP14. Our results showed that ectopic expression of USP14 decreased K48-linked ubiquitination of ERα but not K63-linked ubiquitination in HEC-1A cells (Fig. 3*G*). Furthermore, different dose of the ectopic expression of USP14 showed a significant reduction of K48-linked ubiquitination of ERα in a dose-dependent manner in HEC-1A cells (Fig. 3*H*). Above all, our results suggested that USP14 was involved in ERα deubiquitination with K48-linkage to maintain the stability of ERα.

### USP14 promotes the transcription level of ERα target genes

**Figure 4:**
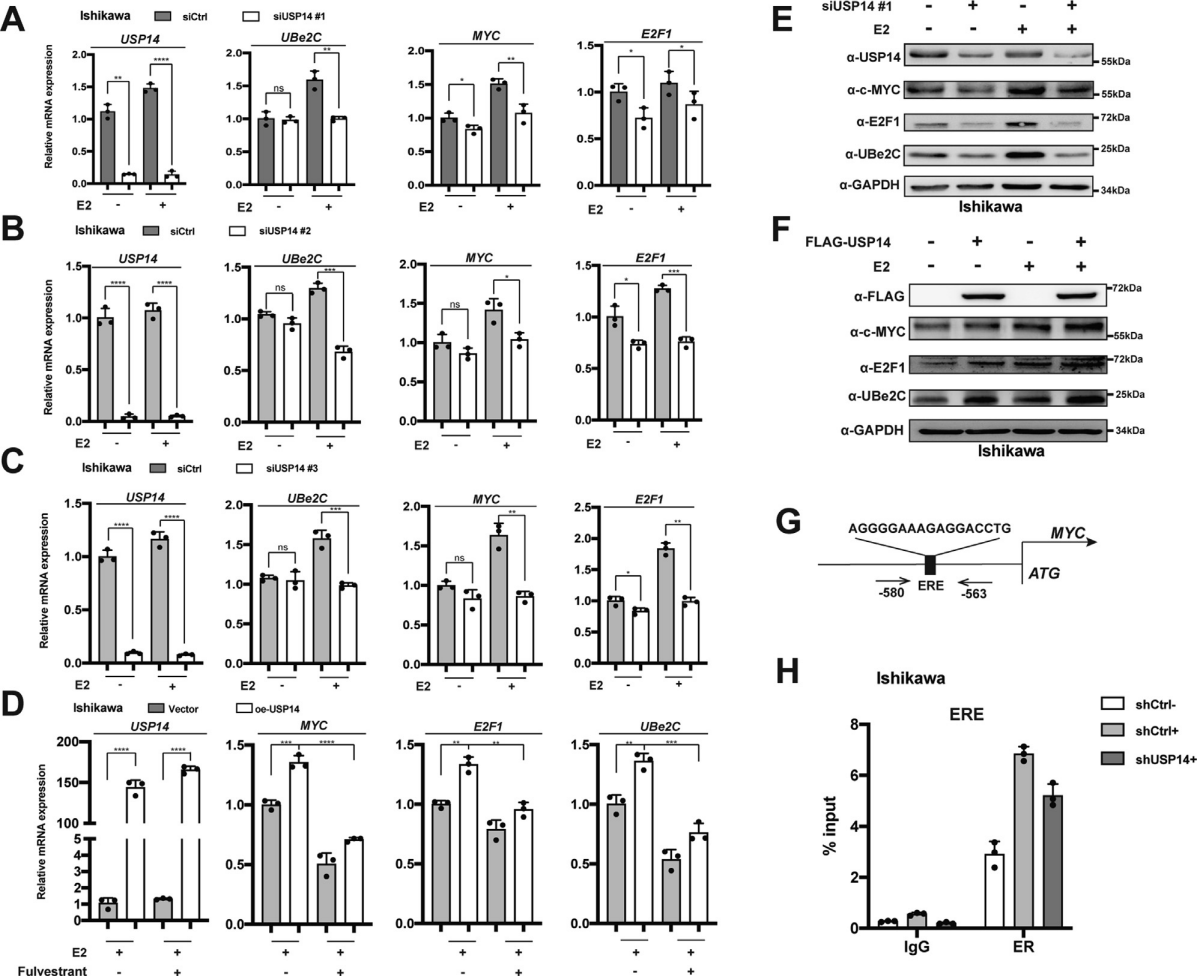
**USP14 promotes the expression of ERα target genes and enhances the recruitment of ERα to ERE region of ERα target gene.***A*–*C*, the effect of USP14 on the mRNA level of endogenous ERα target genes with three independent siRNAs against USP14 (siUSP14 #1, 2, and 3). *D*, the effect of ectopic USP14 expression on mRNA level of endogenous ERα target genes with the treatment of fulvestrant and E2 as indicated. *E* and *F*, the effect of USP14 on the protein level of endogenous ERα target genes with 10 nM E2. *G*, the schematic diagram of the ERα-binding site on the promoter region of *MYC*. *H*, the recruitment of ERα on ERE with the depletion of USP14 in Ishikawa cells. E2, unopposed estrogen; ERα, estrogen receptor α; ERE, estrogen-response element; USP14, ubiquitin-specific peptidase 14.

Having established that USP14 may increase the accumulation of ERα, we then turn to study whether the downstream genes of ERα were modulated by USP14 in EC-derived cells. Quantitative PCR (qPCR) was performed to detect the mRNA expression level of ERα target genes. We examined the mRNA level of *MYC*, *E2F1*, and *UBe2C* in EC-derived cell lines with knockdown of USP14. Our results showed that depletion of USP14 carrying three different sequences of siUPS14 (siUSP14 #1, #2, and #3) obviously inhibited the expression of ERα-regulated genes in the presence of E2 in Ishikawa cells (Fig. 4, *A–C*). To further confirm this result, the Ishikawa cells were transfected with USP14 expression plasmid with cotreatment of 10 nM fulvestrant (antiestrogen) as indicated. The result showed that the upregulation of ectopic USP14 expression on ERα-regulated genes was significantly reduced by fulvestrant treatment (Fig. 4*D*). We further investigated the protein expression levels of ERα downstream genes by Western blot, and our data demonstrated that depletion of USP14 decreased the protein expression levels of ERα-regulated genes. While ectopic expression of USP14 increased these protein expressions of ERα-regulated genes as indicated in EC-derived cells (Fig. 4, *E* and *F*). We then tried to detect whether USP14 had any impact on the recruitment of ERα on the promoter region of ERα target gene. With JASPAR database, we tried to search the putative ERE, which contains the sequence of AGGGGAAAGAGGACCTG on the promoter region of ERα-regulated gene, *MYC*, as indicated (Fig. 4*G*). Chromatin immunoprecipitation (ChIP) assay was then performed to determine the recruitment of ERα at the ERE in the Ishikawa cells carrying shRNA against USP14 (shUSP14). The depletion of USP14 was shown to decrease the recruitment of ERα on the ERE region of *MYC* gene (Fig. 4*H*). Taken together, USP14 was involved in promotion of the transcription of ERα-regulated genes, and USP14 also increased the recruitment of ERα on the promoter region of ERα-regulated gene.

### USP14 promotes the cell proliferation and migration in EC-derived cell lines

**Figure 5:**
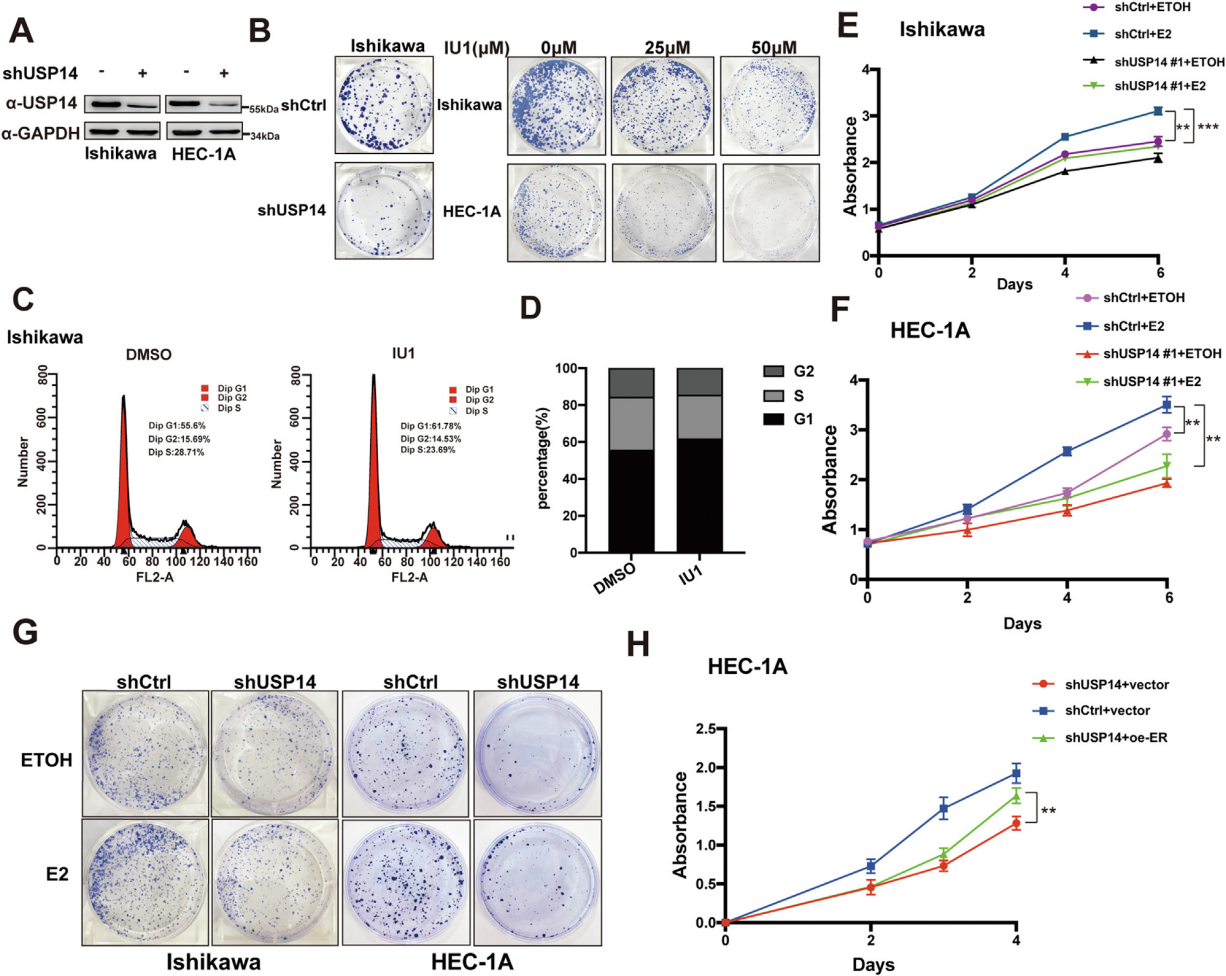
**USP14 promotes the cell proliferation, partly depending on ERα.***A*, the confirmation of USP14 knockdown efficiency in EC cell lines. *B*, the effect of USP14 on cell growth by colony formation assay. The concentration of E2 was 10 nM. *C* and *D*, flow cytometry was performed on Ishikawa cells treated with indicated IU1 for cell cycle analysis. *E* and *F*, depletion of USP14 could inhibit cell proliferation under the stimulation of 10 nM E2 in EC cell lines. *G*, depletion of USP14 could inhibit cell colony formation under the stimulation of E2 in EC cell lines. *H*, the inhibition function of USP14 depletion could be partly reversed by ectopic ERα overexpression. E2, unopposed estrogen; EC, endometrial cancer; ERα, estrogen receptor α; USP14, ubiquitin-specific peptidase 14.

To further examine the function of USP14 in EC-derived cells, we constructed the stable knockdown of USP14 in Ishikawa and HEC-1A cells carrying shUSP14. The efficiency of USP14 knockdown by shUSP14 in EC-derived cell lines was detected with Western blot (Fig. 5*A*). Then colony formation assay was performed in Ishikawa cells, which showed that the depletion of USP14 could inhibit the ability of colony formation. Moreover, Ishikawa cells and HEC-1A cells were treated with different concentrations of USP14-specific inhibitor, IU1 (0, 25, and 50 μM). The results showed that IU1 inhibited the colony formation in a dose-dependent manner in EC-derived cell lines (Figs. 5*B* and S3, *A* and *B*). We then further determined whether USP14 influences the cell cycle, and the flow cytometry analysis was performed in Ishikawa cells with indicated IU1. The results indicated that IU1 could induce G0/G1 arrest (Fig. 5, *C* and *D*). To study the effect of USP14 on the cell proliferation in EC-derived cells, MTS (3-(4,5-dimethylthiazol-2-yl)-5-(3-carboxymethoxyphenyl)-2-(4-sulfophenyl)-2H-tetrazolium) and colony formation assay were performed in the absence or the presence of E2 in Ishikawa and HEC-1A cells. Our data demonstrated that USP14 obviously promoted cell proliferation in EC-derived cell lines (Figs. 5, *E–G* and S3, *C* and *D*). In addition, to determine whether ERα is required for the biological function of USP14 on cell growth in EC-derived cells, overexpression plasmid of ERα was transfected into the Ishikawa cells carrying shUSP14. Our results demonstrated that ectopic expression of ERα could partially reverse the growth inhibition caused by shUSP14 (Fig. 5*H*). Taken together, USP14 is involved in promotion of cell growth in EC-derived cells at least partially *via* E2–ERα.

**Figure 6:**
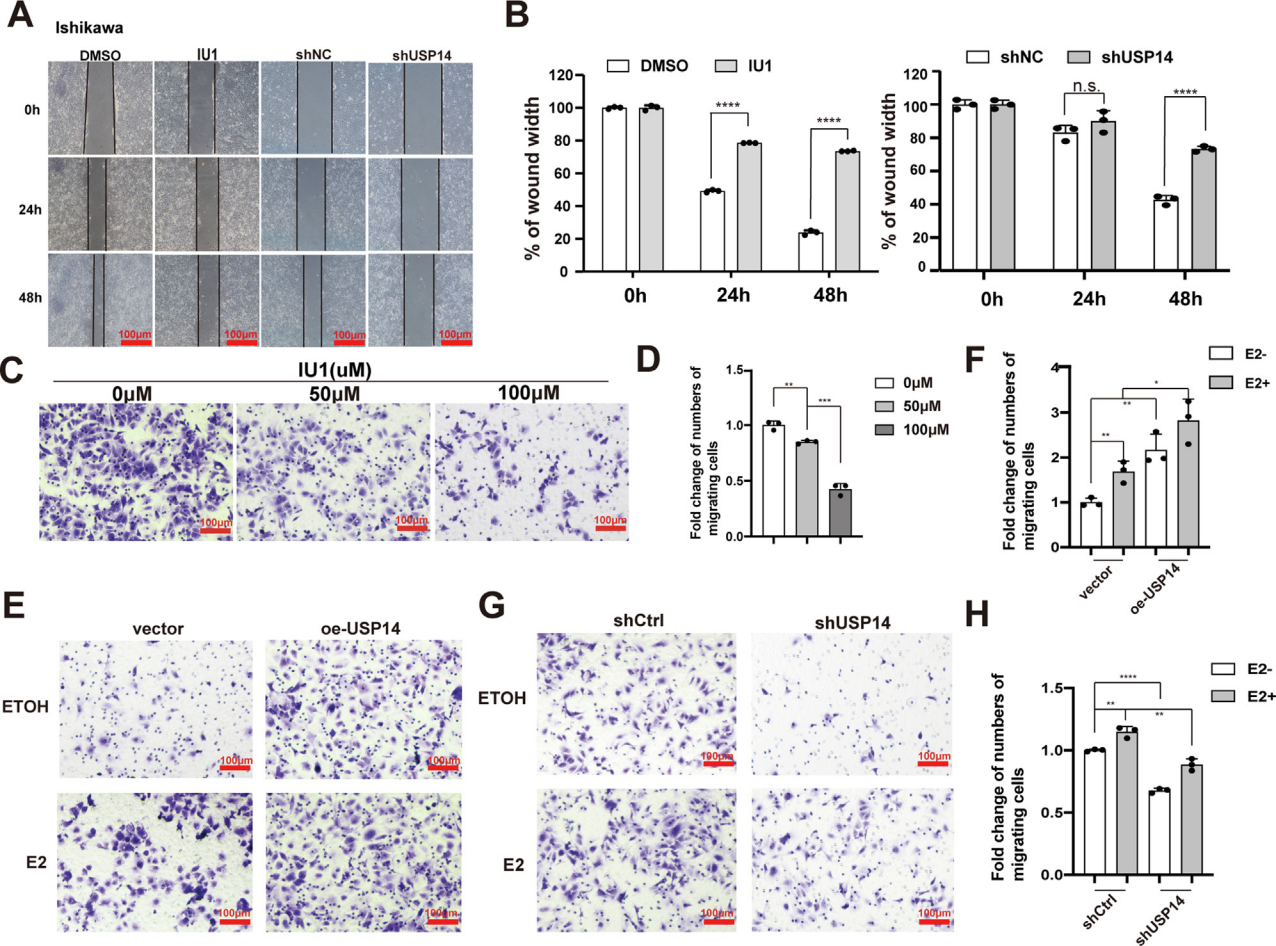
**USP14 facilities the cell migration *via* ERα.***A* and *B*, USP14 depletion or USP14 inhibition (IU1) could inhibit cell migration by wound healing assay in Ishikawa cells. The scale bars represent 100 μm. *C* and *D*, transwell assay was performed with IU1 as indicated. IU1 could inhibit cell migration in a dose-dependent manner. The scale bars represent 100 μm. *E* and *F*, overexpression of USP14 (oe-USP14) promoted cell migration in the presence of E2. Three independent experiments were statistically analyzed as indicated. The scale bars represent 100 μm. *G* and *H*, depletion of USP14 could suppress cell migration in the presence of E2. Three independent experiments were statistically analyzed as indicated. The scale bars represent 100 μm. ∗*p* < 0.05, ∗∗*p* < 0.01, ∗∗∗*p* < 0.001, and ∗∗∗∗*p* < 0.0001. E2, unopposed estrogen; ERα, estrogen receptor α; USP14, ubiquitin-specific peptidase 14.

To investigate the effect of USP14 on cell migration, the wound healing assay was performed in Ishikawa cells carrying shUSP14 or IU1 treatment. The results showed that inhibition of USP14 significantly decreased the cell migration in Ishikawa cells (Fig. 6, *A* and *B*). Furthermore, with different concentrations of IU1 treatment, the results from the transwell assay showed that the migration ability of Ishikawa cells was inhibited by IU1 treatment in a dose-dependent manner (Fig. 6, *C* and *D*). Subsequently, the transwell assay was further performed with Ishikawa cells under the treatment of E2. The results demonstrated that USP14 depletion inhibited cell migration, and conversely, ectopic expression of USP14 promoted cell migration in the presence of E2 (Fig. 6, *E–H*).

### Depletion of USP14 or USP14-specific inhibitor suppresses cell growth in EC-derived cell lines and in mice

**Figure 7:**
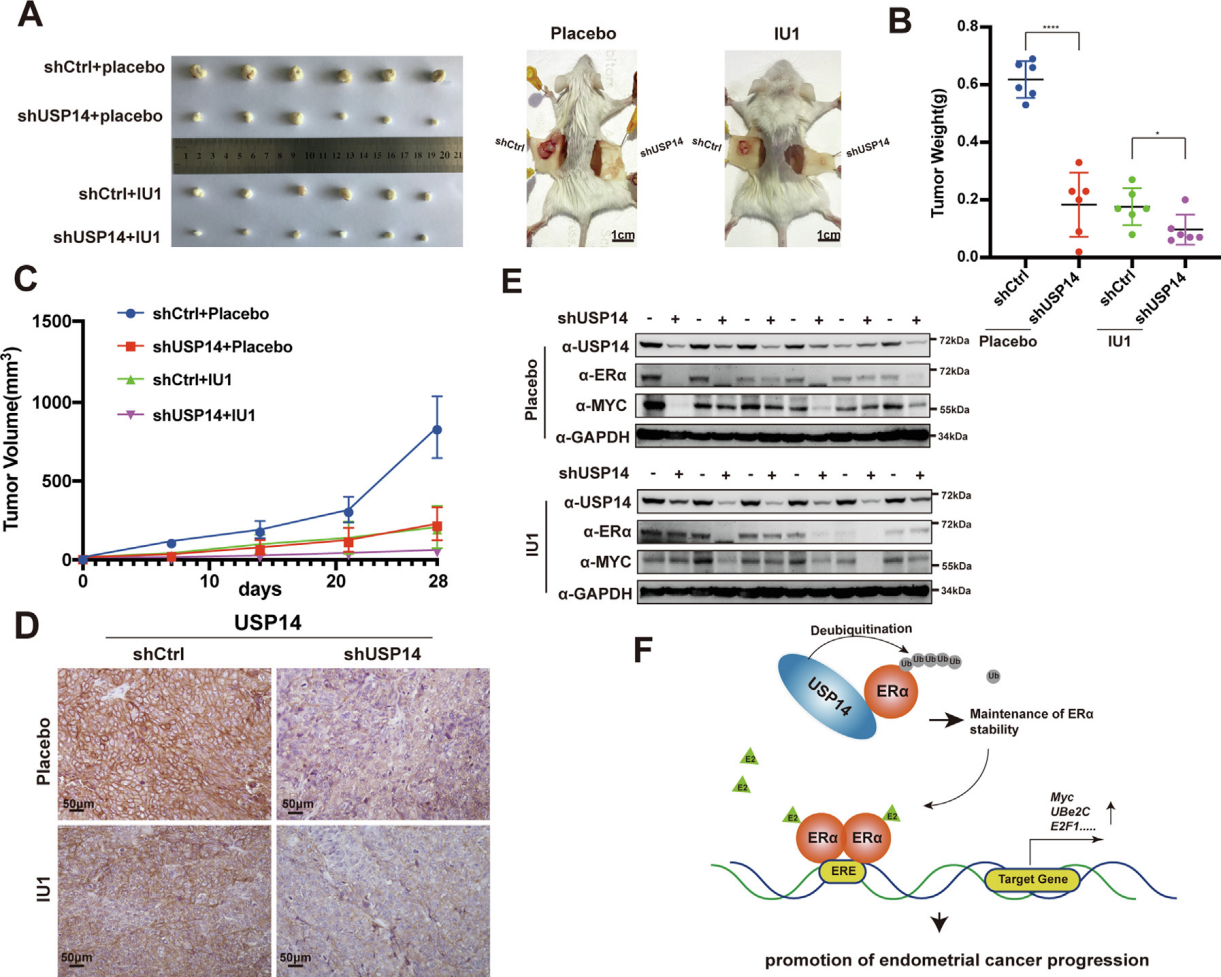
**Depletion or inhibition of USP14 can suppress cell growth in mice.***A*, photos were shown to representative the tumor bearing as indicated. The scale bars represent 1 cm. *B* and *C*, tumor weight and volume of different groups were shown as indicated. *D*, immunohistochemical assay was performed to detect the expression of USP14 in the xenograft tumor tissues. The scale bars represent 50 μm. *E*, protein level of USP14 in every xenograft tumor tissue was detected by Western blot. *F*, schematic diagram was presented to demonstrate the interaction between USP14 and ERα and the function of USP14 in the development of EC. EC, endometrial cancer; ERα, estrogen receptor α; USP14, ubiquitin-specific peptidase 14.

To further examine the biological function of USP14 in cell growth *in vivo*, we conducted subcutaneous xenograft tumor experiments in nonobese diabetic/severe combined immunodeficiency mice to detect the effect of USP14 depletion on cell growth in EC-derived cells. The Ishikawa cells (1 × 10^7^ cells/mouse) transfected with shCtrl or shUSP14 were, respectively, injected into the left and right flanks of the 4-week-old female mice. The results demonstrated that the combination of shUSP14 and IU1 appeared to show the best antitumor activity. The tumors with shUSP14 or treated with IU1 alone were similar in size (Fig. 7*A*). The tumor weight and volume significantly declined under the treatment of shUSP14 or IU1 alone and declined more obviously under the combined treatment of shUSP14 and IU1 (Fig. 7, *B* and *C*). Immunohistochemical (IHC) experiments were then performed to detect the expression level of USP14 in xenograft tumor derived from Ishikawa cells (Fig. 7*D*). In addition, Western blot was performed to examine the protein level of USP14, ERα, and c-Myc in the above xenograft tumor. The results further demonstrated that USP14 depletion significantly decreased ERα and c-Myc expression (Fig. 7*E*). Taken together, our results suggest that knockdown of USP14 or IU1 treatment inhibits EC-derived cell growth in mice.

## Discussion

EC is one of the common carcinomas in women’s reproductive system. The treatment of hysterectomy is efficacious in patients at an early stage. However, the intervention strategy for the relapse patients and the advanced stage patients is limited. Although EC belongs to one of the hormone-dependent tumors, the endocrine therapy in EC is not effective compared with that in BCa. It has been considered that E2–ERα signaling pathway is essential for the development of EC. However, the biological function of ERα coregulator in EC is still elusive. In this study, our results have demonstrated that USP14 is involved in the maintenance of ERα stabilization to contribute to the progression of EC, providing a potential therapeutic target for EC patients, especially for fertility preservation of EC patients (Fig. 7*F*).

It has been reported that USP14 is highly related to cancer occurrence and progression in various kinds of carcinomas. As one of the proteasome-associated deubiquitinating enzymes, USP14 has a dual function in protein degradation and proteasome function. USP14 is elevated in prostate cancer and AR-positive BCa, and the expression of USP14 is positively correlated with that of AR. Downregulation of USP14 may suppress cell proliferation, migration, and promote cell apoptosis *via* stabilizing AR in prostate cancer or AR-positive BCa (16, 25). In addition, USP14 enhances Wnt/β-catenin signaling pathway *via* stabilizing β-catenin to promote tumor development in lung cancer and hepatocellular cancer (26, 27). However, the biological function and underlying molecular mechanism of USP14 in EC progression is still unclear. Previous study has indicated that USP14 is a predictor of recurrence for EC patients, and pharmacological inhibition of USP14 with VXL1570 may increase the EC patients’ chemotherapeutic response rate (28). Similar biological function results are found in our study. Our results suggest that USP14 is upregulated in EC clinical samples, and the high expression of USP14 is positively correlated with the poor prognosis of EC patients. Different from previous studies, our study focuses more on providing insight into the relationship between USP14 and E2–ERα, which is a main risk factor for the initiation of EC. Our study has demonstrated that USP14 is involved in the maintenance of ERα through ERα deubiquitination. Moreover, we also provide the evidence that USP14 depletion or USP14 inhibitor treatment abrogates cell growth in EC-derived cell lines and mice, suggesting that the interaction between USP14 and ERα may promote EC progression.

Unopposed estrogen exposure is one of the main risk factors for type I EC. It has been putatively considered that almost 80% of type I EC is ERα positive, indicating that modulation of E2–ERα signaling pathway would be essential for the development of ERα-related cancers, such as BCa and EC. It is well known that ERα protein stability is strictly controlled by ubiquitin proteasome system. Previously, studies have established that E3 ligases, such as RNF8 and TRIM11, may modulate ERα monoubiquitination to stabilize ERα and then promote BCa progression (9, 29). USP22 has been identified to deubiquitinate ERα with both K48-linkage and K63-linkage to enhance the stability of ERα, thereby promoting cell growth and endocrine resistance in BCa (30). As a deubiquitinase, UCH-L1 suppresses ERα transcription through the deubiquitinase-mediated stability of epidermal growth factor receptor, which is a corepressor of ERα (10). Thus, understanding well the modulation of ERα in ERα-related tumors would provide the potential therapeutic strategy for tumor treatment. In this study, our results have shown that USP14 associates with ERα to maintain the stability of ERα *via* its deubiquitination activity in EC. USP14 depletion or USP14 inhibitor treatment enhances ERα polyubiquitination level with K48-linkage but not K63-linkage. Our data suggest that USP14 involved in the enhancement of ERα stability in EC may be a new strategy for developing the endocrine therapeutic target for EC treatment.

Our previous study has demonstrated that Ash2L acting as a coactivator of ERα is associated with MLL1–WDR5 complex to enhance ERα action and participate in the promotion of EC progression (31). In addition, it has been reported that mutations in the ligand-binding domain of ERα may cause continuous activation of the ERα signaling pathway in EC (32). Our data have demonstrated that USP14 enhances the recruitment of ERα on the ERE sites of ERα-regulated genes, thereby increasing the transcription of these genes, such as *MYC*, *UBe2C*, and so on (Fig. 4). *UBe2C* has been identified as an ERα target gene to promote epithelial–mesenchymal transition in EC (33). In concordance with this, our results have shown that *UBe2C* was also upregulated by USP14 in EC. Taken together, our results suggest that USP14 might act as the coregulator of ERα, promoting the development of EC.

In summary, this study has demonstrated that USP14 associates with ERα to maintain the stability of ERα through its deubiquitination activity with the K48-linkage polychain. USP14 leads to an enhancement of ERα recruitment on ERE regions of ERα downstream target genes, thereby upregulating ERα action. Moreover, depletion of USP14 or USP14-specific inhibitor treatment inhibits cell proliferation and migration in EC, indicating that USP14 may be a novel therapeutic target in EC treatment.

## Experimental procedures

### Cell culture and reagents

The human EC cell lines Ishikawa and HEC-1A were cultured in RPMI1640 (Gibco-BRL). Human embryonic kidney 293 and COS7 cells were cultured in Dulbecco’s modified Eagle’s medium (Gibco-BRL). All the culture media contained 10% fetal bovine serum, penicillin (100 U/ml), and streptomycin (100 ng/ml) at 37 °C with 5% CO_2_ in the incubator.

USP14 inhibitor, IU1, and cycloheximide were ordered from AbMole. MTS was purchased from Promega. 17β-estradiol was obtained from Sigma. MG132 was bought from Selleck (CAS no.: 133407-82-6).

### Antibodies

The antibodies in our research were anti-ERα (Cell Signaling Technology; catalog no.: 8644), anti-GAPDH (Shanghai Kangchen; catalog no.: KC5G4), anti-His (Proteintech; catalog no.: 66005-1-lg), anti-USP14 (Santa; catalog no.: sc-398099), anti-USP14 (Bethyl; catalog no.: A300-919A), anti-Ub (Proteintech; catalog no.: 10210-2-AP), anti-rabbit/mouse (ABclonal), anti-MYC (Proteintech; catalog no.: 10828-1-AP), anti-E2F1 (Proteintech; catalog no.: 66515-1-lg), anti-UBe2C (Proteintech; catalog no.: 66087-1-lg), and anti-immunoglobulin G (Santa; catalog no.: sc-2025).

### Co-IP and Western blot analysis

For exogenous co-IP, COS7 cells were transfected with FLAG-USP14 and HA-ERα with jetPRIME DNA transfection reagent (Polyplus); after 4 to 6 h, the cells were treated with E2 for 48 h. The whole-cell lysates were extracted after 48 h. The lysates were incubated with anti-HA for 12 h and then with protein G Sepharose for 4 h. The immune complexes were detected with indicated antibodies. Endogenic co-IP was similar as the aforementioned procedures.

Western blot analysis was performed by the standard process introduced in our previous study (34).

### RNA isolation and real-time qPCR

Cells transfected with siCtrl or siUSP14 for 24 h were prepared for RNA isolation. Total RNA was extracted with Trizol (Vazyme), and complementary DNA was reversed using PrimeScript RT–PCR Kit. Then qPCR was performed with SYBR qPCR Master Mix (Vazyme) on LightCycler96 (Roche). All the primers used in our experiment were showed in Table 1.

**Table 1:** The primer sequences for the indicated genes used in qRT–PCR

Name	Sense (F′)	Antisense (R′)
18S	TTGACGGAAGGGCACCACCA	GCACCACCACCCACGGAAT
USP14	TGTGCCTGAACTCAAAGATGC	ATATACTGCGCTGAAGCCATTT
ESR1	CTAACTTGCTCTTGGACAGGA	CAGGACTCGGTGGATATGGT
Myc	CGTCCTCGGATTCTCTGCTC	GCTGGTGCATTTTCGGTTGT
UBe2C	GGATTTCTGCCTTCCCTGAA	GATAGCAGGGCGTGAGGAAC
E2F1	CAGAGCAGATGGTTATGGTGA	AGATGATGGTGGTGGTGACA

### siRNA transfection and lentiviral infection

siRNA was purchased from Sigma, and the shUSP14 lentivirus was bought from GeneChem. EC cells were transfected with them according to the protocol.

### ChIP assay

ChIP assay was performed similar to our previous study (35). Ishikawa cells were collected for ChIP assay after treated with E2 for 12 h. Immunoprecipitation of sonicated chromatin solutions was conducted by overnight incubation at 4 °C by rotating with anti-ERα or anti-USP14. Protein A was added to every sample the next day, and then the samples were washed with low salt buffer, high salt buffer, lithium chloride buffer, and Tris–EDTA buffer. Then the complexes were eluted and purified. The DNA samples were then amplified by qPCR. The specific primers of *c-Myc* were sense: 5′-TTGACGGAAGGGCACCACCA-3′, antisense: 5′-GCACCACCACCCACGGAAT-3’.

### Cell growth, colony formation assay, and transwell assay

For cell growth analysis, 4 × 10^3^ Ishikawa or HEC-1A cells were plated in 96-well plate and stimulated with 10^−8^ M E2 or absolute ethanol. The cells were collected at indicated days and measured at an absorbance of 490 nm.

Colony formation assay was performed with Ishikawa and HEC-1A cells cultured with fetal bovine serum RPMI1640. The cells were cultured in an atmosphere of 5% CO_2_ at 37 °C for 14 days. PBS was used to wash the cells, and then 4% polyformaldehyde was added into the plate to fix the cells for 20 min. After that, the cells were stained with 1% crystal violet for 1 h or overnight.

Transwell assay was performed with Ishikawa cells. About 3 ×b10^4^ cells were seeded in the transwell chambers with the treatment of different concentrations of IU1. The indicated concentration of E2 was 10^−8^ M. The cells were collected after 24 h.

### IHC assay

Paraffin sections (5 μm thick) of benign endometrial tissues (n = 31) and EC tissue samples (n = 99) were deparaffinized, rehydrated, and antigen retrieval performed. After blocked with donkey serum overnight, the slides were incubated with rabbit anti-USP14 (1:2000 dilution) antibodies (36, 37). After washed in PBS, the sections were incubated with goat anti-rabbit immunoglobulin G for 1 h at room temperature, followed by visualization with diaminobenzidine of the expression of USP14 and visualization with hematoxylin of the nuclei. All the images were captured by an Olympus microscope. The samples were approved by the Human Research Ethics Committee of China Medical University.

### Xenograft tumor growth

Nonobese diabetic/severe combined immunodeficiency mice were purchased from Vital River Laboratory. All the animal experiments were approved by the China Medical University of Animal Care Center. Ishikawa cells (1.0 × 10^7^ cells/mouse) stably expressing shCtrl or shUSP14 were subcutaneously inoculated into the 5-week-old female mice for each side. The mice were divided into two groups: one group was treated with placebo and the other group was orally treated with IU1 (40 mg/kg/day) every 2 days. Tumor diameter was measured every week. Tumor volume (mm^3^) was calculated as volume = (short diameter)^2^ × (long diameter)/2 (38).

### Patient samples

Human normal endometrium and EC tissues were obtained from the First Hospital of China Medical University. All the EC samples we used for IHC assay are endometrioid adenocarcinoma, including stage I (86 cases [86.9%]), stage II (7 cases [7%]), and stage III (6 cases [6.1%]). And all the samples were under patients’ informed contents.

### Statistics

Unpaired Student’s *t* test was used to examine the statistical significance. Pearson correlation coefficient was used to test the expression level of USP14 and ERα. The overall survival and relapse-free survival were calculated with Kaplan–Meier plotter. *p* Value was calculated with GraphPad Prism 8 (GraphPad Software, Inc), and less than 0.05 was considered statistically significant.

## Data availability

The data are included in the main file and supporting information.

## Supporting information

This article contains supporting information (20).

## Conflict of interest

The authors declare that they have no conflicts of interest with the contents of this article.

## References

[1] Ferlay J., Soerjomataram I., Dikshit R., Eser S., Mathers C., Rebelo M. (2015). Cancer incidence and mortality worldwide: sources, methods and major patterns in GLOBOCAN 2012. Int. J. Cancer.

[2] Bray F., Ferlay J., Soerjomataram I., Siegel R.L., Torre L.A., Jemal A. (2018). Global cancer statistics 2018: GLOBOCAN estimates of incidence and mortality worldwide for 36 cancers in 185 countries. CA Cancer J. Clin..

[3] Arend R.C., Jones B.A., Martinez A., Goodfellow P. (2018). Endometrial cancer: molecular markers and management of advanced stage disease. Gynecol. Oncol..

[4] Siegel R.L., Miller K.D., Fuchs H.E., Jemal A. (2021). Cancer statistics, 2021. CA Cancer J. Clin..

[5] Bokhman J.V. (1983). Two pathogenetic types of endometrial carcinoma. Gynecol. Oncol..

[6] Carlson M.J., Thiel K.W., Leslie K.K. (2014). Past, present, and future of hormonal therapy in recurrent endometrial cancer. Int. J. Womens Health.

[7] Tecalco-Cruz A.C., Ramirez-Jarquin J.O., Cruz-Ramos E. (2019). Estrogen receptor alpha and its ubiquitination in breast cancer cells. Curr. Drug Targets.

[8] Xia X., Liao Y., Huang C., Liu Y., He J., Shao Z. (2019). Deubiquitination and stabilization of estrogen receptor alpha by ubiquitin-specific protease 7 promotes breast tumorigenesis. Cancer Lett..

[9] Tang J., Luo Y., Tian Z., Liao X., Cui Q., Yang Q. (2020). TRIM11 promotes breast cancer cell proliferation by stabilizing estrogen receptor alpha. Neoplasia.

[10] Chen X.S., Wang K.S., Guo W., Li L.Y., Yu P., Sun X.Y. (2020). UCH-L1-mediated down-regulation of estrogen receptor alpha contributes to insensitivity to endocrine therapy for breast cancer. Theranostics.

[11] Yu F., Liu J.B., Wu Z.J., Xie W.T., Zhong X.J., Hou L.K. (2018). Tumor suppressive microRNA-124a inhibits stemness and enhances gefitinib sensitivity of non-small cell lung cancer cells by targeting ubiquitin-specific protease 14. Cancer Lett..

[12] Sharma A., Almasan A. (2020). USP14 regulates DNA damage response and is a target for radiosensitization in non-small cell lung cancer. Int. J. Mol. Sci..

[13] Liu B., Jiang S., Li M., Xiong X., Zhu M., Li D. (2018). Proteome-wide analysis of USP14 substrates revealed its role in hepatosteatosis via stabilization of FASN. Nat. Commun..

[14] Kim H.T., Goldberg A.L. (2018). UBL domain of Usp14 and other proteins stimulates proteasome activities and protein degradation in cells. Proc. Natl. Acad. Sci. U. S. A..

[15] Hanna J., Hathaway N.A., Tone Y., Crosas B., Elsasser S., Kirkpatrick D.S. (2006). Deubiquitinating enzyme Ubp6 functions noncatalytically to delay proteasomal degradation. Cell.

[16] Liao Y., Xia X., Liu N., Cai J., Guo Z., Li Y. (2018). Growth arrest and apoptosis induction in androgen receptor-positive human breast cancer cells by inhibition of USP14-mediated androgen receptor deubiquitination. Oncogene.

[17] Xu L., Wang J., Yuan X., Yang S., Xu X., Li K. (2020). IU1 suppresses proliferation of cervical cancer cells through MDM2 degradation. Int. J. Biol. Sci..

[18] Shen J., Hong L., Chen L. (2020). Ubiquitin-specific protease 14 regulates ovarian cancer cisplatin-resistance by stabilizing BCL6 oncoprotein. Biochem. Biophysical Res. Commun..

[19] Wang D., Ma H., Zhao Y., Zhao J. (2021). Ubiquitin-specific protease 14 is a new therapeutic target for the treatment of diseases. J. Cell Physiol..

[20] Chandrashekar D.S., Bashel B., Balasubramanya S.A.H., Creighton C.J., Ponce-Rodriguez I., Chakravarthi B. (2017). Ualcan: a portal for facilitating tumor subgroup gene expression and survival analyses. Neoplasia.

[21] Nagy A., Munkacsy G., Gyorffy B. (2021). Pancancer survival analysis of cancer hallmark genes. Sci. Rep..

[22] Lee B.H., Lee M.J., Park S., Oh D.C., Elsasser S., Chen P.C. (2010). Enhancement of proteasome activity by a small-molecule inhibitor of USP14. Nature.

[23] Kravtsova-Ivantsiv Y., Sommer T., Ciechanover A. (2013). The lysine48-based polyubiquitin chain proteasomal signal: not a single child anymore. Angew. Chem. Int. Ed. Engl..

[24] Kim H.T., Kim K.P., Lledias F., Kisselev A.F., Scaglione K.M., Skowyra D. (2007). Certain pairs of ubiquitin-conjugating enzymes (E2s) and ubiquitin-protein ligases (E3s) synthesize nondegradable forked ubiquitin chains containing all possible isopeptide linkages. J. Biol. Chem..

[25] Liao Y., Liu N., Hua X., Cai J., Xia X., Wang X. (2017). Proteasome-associated deubiquitinase ubiquitin-specific protease 14 regulates prostate cancer proliferation by deubiquitinating and stabilizing androgen receptor. Cell Death Dis..

[26] Huang G., Li L., Zhou W. (2015). USP14 activation promotes tumor progression in hepatocellular carcinoma. Oncol. Rep..

[27] Wu N., Liu C., Bai C., Han Y.P., Cho W.C., Li Q. (2013). Over-expression of deubiquitinating enzyme USP14 in lung adenocarcinoma promotes proliferation through the accumulation of beta-catenin. Int. J. Mol. Sci..

[28] Vogel R.I., Pulver T., Heilmann W., Mooneyham A., Mullany S., Zhao X. (2016). USP14 is a predictor of recurrence in endometrial cancer and a molecular target for endometrial cancer treatment. Oncotarget.

[29] Wang S., Luo H., Wang C., Sun H., Sun G., Sun N. (2017). RNF8 identified as a co-activator of estrogen receptor alpha promotes cell growth in breast cancer. Biochim. Biophys. Acta Mol. Basis Dis..

[30] Wang S., Zhong X., Wang C., Luo H., Lin L., Sun H. (2020). USP22 positively modulates ERalpha action via its deubiquitinase activity in breast cancer. Cell Death Differ..

[31] Zeng K., Wu Y., Wang C., Wang S., Sun H., Zou R. (2020). ASH2L is involved in promotion of endometrial cancer progression via upregulation of PAX2 transcription. Cancer Sci..

[32] Backes F.J., Walker C.J., Goodfellow P.J., Hade E.M., Agarwal G., Mutch D. (2016). Estrogen receptor-alpha as a predictive biomarker in endometrioid endometrial cancer. Gynecol. Oncol..

[33] Liu Y., Zhao R., Chi S., Zhang W., Xiao C., Zhou X. (2020). UBE2C is upregulated by estrogen and promotes epithelial-mesenchymal transition via p53 in endometrial cancer. Mol. Cancer Res..

[34] Shi W., Yan D., Zhao C., Xiao M., Wang Y., Ma H. (2017). Inhibition of IL-6/STAT3 signaling in human cancer cells using Evista. Biochem. Biophys. Res. Commun..

[35] Sun S., Zhong X., Wang C., Sun H., Wang S., Zhou T. (2016). BAP18 coactivates androgen receptor action and promotes prostate cancer progression. Nucleic Acids Res..

[36] Lv C., Wang S., Lin L., Wang C., Zeng K., Meng Y. (2021). USP14 maintains HIF1-alpha stabilization via its deubiquitination activity in hepatocellular carcinoma. Cell Death Dis..

[37] Kim H.T., Goldberg A.L. (2017). The deubiquitinating enzyme Usp14 allosterically inhibits multiple proteasomal activities and ubiquitin-independent proteolysis. J. Biol. Chem..

[38] Zhang X., Kyo S., Nakamura M., Mizumoto Y., Maida Y., Bono Y. (2014). Imatinib sensitizes endometrial cancer cells to cisplatin by targeting CD117-positive growth-competent cells. Cancer Lett..

