# Supplementary material for: Ubiquitin-specific peptidase 14 maintains estrogen receptor α stability via its deubiquitination activity in endometrial cancer
Source: J Biol Chem. 2022 Nov 22;299(1):102734. doi: 10.1016/j.jbc.2022.102734 (PMC9800553; doi:10.1016/j.jbc.2022.102734)
Supplement: Supplemental Figures S1–S3 [file mmc1.pdf]

Supporting Information

Ubiquitin-specific peptidase 14 maintains estrogen receptor α stability via its deubiquitination activity in endometrial cancer

Yingjie Su[1,2], Kai Zeng[1], Shuchang Liu[3], Yi Wu[1,4], Chunyu Wang[1], Shengli Wang[1], Lin Lin[1], Renlong Zou[1], Ge Sun[1], Ruina Luan[1], Baosheng Zhou[1], Yu Bai[1], Jumin Niu[5], Yi Zhang [2]*, and Yue Zhao[1]*

[1]Department of Cell Biology, Key laboratory of Cell Biology, Ministry of Public Health, and Key laboratory of Medical Cell Biology, Ministry of Education, School of Life Sciences, China Medical University, Shenyang City, Liaoning Province 110122, China

[2]Department of Gynecology, the First Hospital of China Medical University, Shenyang City, Liaoning Province 110001, P.R. China

[3]Department of Gynecology, the Fourth Affiliated Hospital of China Medical University, Shenyang City, Liaoning Province 110032, China

[4]Department of Pathogenic Biology, Shenyang Medical College, Shenyang, Liaoning, 110034, China

[5]Department of Obstetrics and Gynecology, Shenyang Women's and Children's Hospital, Shenyang, Liaoning, 110011, China

Supporting Figures

Figure S1

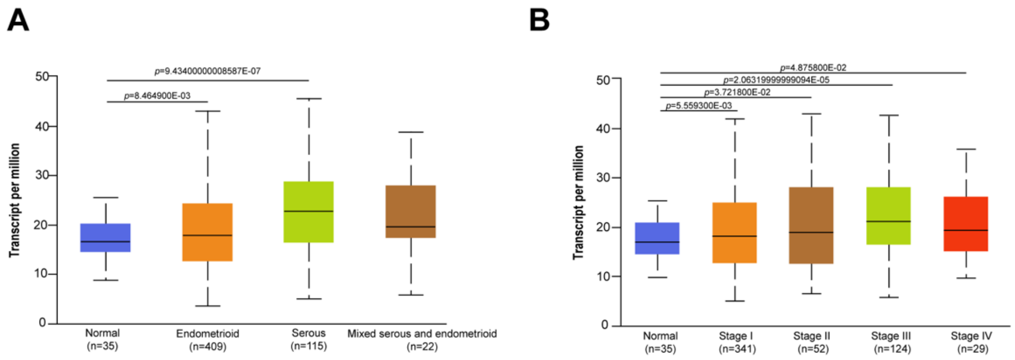

Figure S1. The differential expression of USP14 between normal and cancerous tissues in TCGA database.

(A) The expression of USP14 among different histological subtypes. (B) The differential expression of USP14 between normal tissues and EC patients from Stage I to Stage IV.

Figure S2

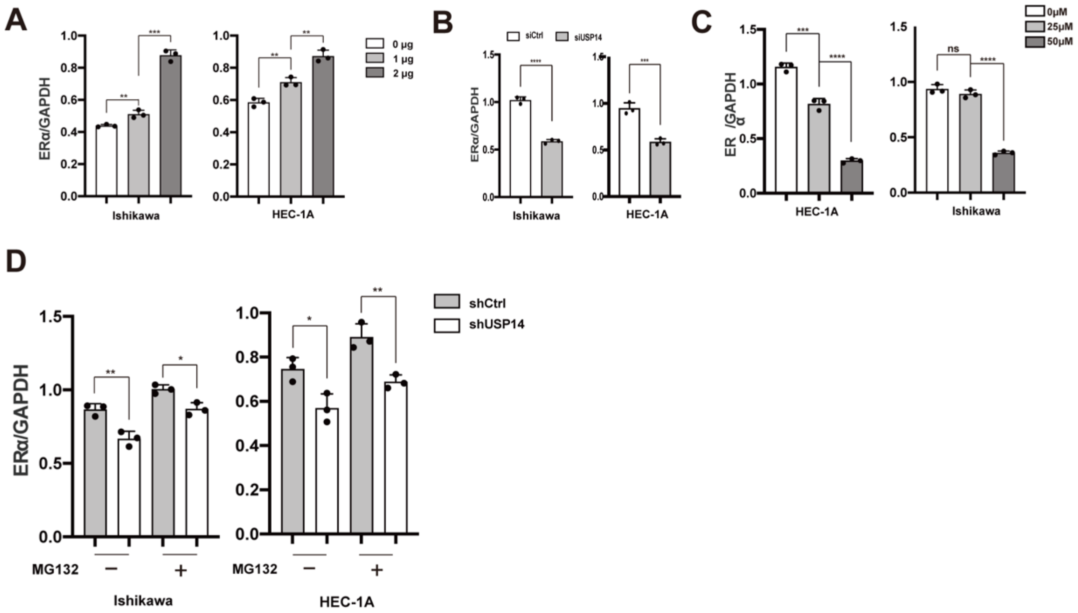

Figure S2. USP14 participates in maintenance of ERα stability

(A) The quantitated expression of ERα protein under different dose of USP14 overexpression in Ishikawa cells and HEC-1A cells. (B) Ishikawa cells and HEC-1A cells were transfected with siRNA of USP14 and the ERα protein expression level was calculated with Image J. (C), ERα protein level decreased in a dose-dependent manner of IU1. The concentration of IU1 was 0 μM, 25 μM and 50 μM. (D) The ERα protein expression level were calculated via Image J in Ishikawa cells and HEC-1A cells carrying shUSP14 treated with MG132. $^{*}P<0.05$, $^{**}P<0.01$, $^{***}P<0.001$, $^{****}P<0.0001$. ns stands for no significance.

Figure S3

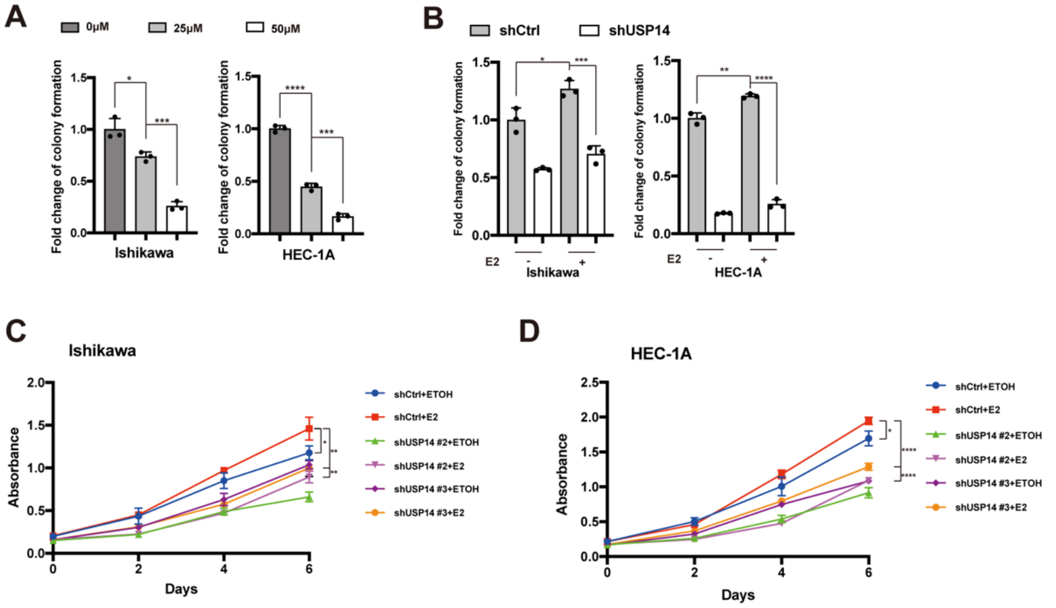

Figure S3. USP14 promotes the cell proliferation under E2 stimulation.

(A) The statistical analysis of colony formation assay under different concentration of IU1 as indicated. (B) The statistical analysis for colony formation assay of both Ishikawa cells and HEC-1A cells were performed to detect the effect of depletion of USP14 with the stimulation of 10nM E2. (C-D) The depletion of USP14 inhibit Ishikawa cells and HEC-1A cells proliferation with 10 nM E2 stimulation. $P<0.05$, $^{**}P<0.01$, $^{***}P<0.001$, $^{****}P<0.0001$. The target sequence of shUSP14 #2 and #3 were from the siUSP14 #2 and #3.
